# Electronic medical records imputation by temporal Generative Adversarial Network

**DOI:** 10.1186/s13040-024-00372-2

**Published:** 2024-06-26

**Authors:** Yunfei Yin, Zheng Yuan, Islam Md Tanvir, Xianjian Bao

**Affiliations:** 1 College of Computer Science, Chongqing University, Chongqing, 400044 China; 2 Department of Computer Science, Maharishi University of Management, Fairfield, IOWA USA

**Keywords:** Electronic medical record, Missing value, Time decay, Generative adversarial networks, Association relation

## Abstract

The loss of electronic medical records has seriously affected the practical application of biomedical data. Therefore, it is a meaningful research effort to effectively fill these lost data. Currently, state-of-the-art methods focus on using Generative Adversarial Networks (GANs) to fill the missing values of electronic medical records, achieving breakthrough progress. However, when facing datasets with high missing rates, the imputation accuracy of these methods sharply deceases. This motivates us to explore the uncertainty of GANs and improve the GAN-based imputation methods. In this paper, the GRUD (Gate Recurrent Unit Decay) network and the UGAN (Uncertainty Generative Adversarial Network) are proposed and organically combined, called UGAN-GRUD. In UGAN-GRUD, it highlights using GAN to generate imputation values and then leveraging GRUD to compensate them. We have designed the UGAN and the GRUD network. The former is employed to learn the distribution pattern and uncertainty of data through the Generator and Discriminator, iteratively. The latter is exploited to compensate the former by leveraging the GRUD based on time decay factor, which can learn the specific temporal relations in electronic medical records. Through experimental research on publicly available biomedical datasets, the results show that UGAN-GRUD outperforms the current state-of-the-art methods, with average 13% RMSE (Root Mean Squared Error) and 24.5% MAPE (Mean Absolute Percentage Error) improvements.

## Introduction

**Fig. 1:**
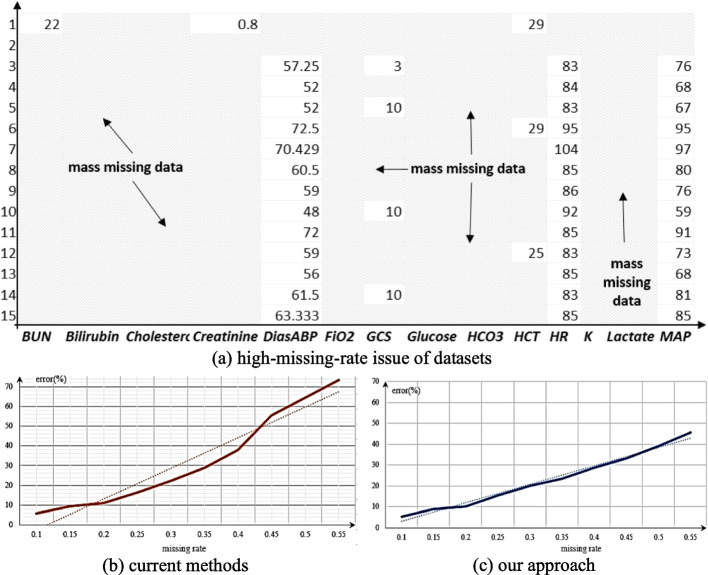
High-missing-rate dataset and imputation methods. For such a high-missing-rate dataset (Fig. 1a), the imputation effect of current GAN-based methods is shown in the bottom-left (Fig. 1b), and the effect of our approach is shown in the bottom-right (Fig. 1c) that can achieve an average improvement of 13.0% RMSE (Root Mean Squared Error) and 24.5% MAPE (Mean Absolute Percentage Error)

Electronic medical records are often lost due to equipment failures, data transmission interruptions, and other reasons [1]. As a result, the final collections of electronic medical records are often sparse and irregular. To fill in the lost values in electronic medical records, most state-of-the-art methods currently employ Generative Adversarial Networks (GANs) [2], which can learn the distribution of the original dataset and generate imputation values. However, when the missing rate of the dataset is high, there is a significant deviation between the learned data distribution by GANs and the actual data distribution, which leads to a sharp decrease in the accuracy of missing value imputation. Figure 1 shows the missing situation of Health-care [3], a publicly available dataset of electronic medicine.

In Fig. 1, there are a total of 42 physiological attributes and 196,000 records. That is to say, In Fig. 1a, *BUN*, *Bilirubin*, *Cholesterol*, *Creatinine*, *DiasABP*, *FiO2*, *GCS*, *Glucose*, *HCO3*, *HCT*, *HR*, *K*, *Lactate*, *MAP* are physiological attributes, and the ordinate represents the serial number of records in the dataset. Obviously, the values of *Bilirubin*, *Cholesterol*, and *HCO3* are the most severely lost. For the high-missing-rate dataset, the imputation errors of the state-of-the-art GAN-based methods are quite high, as shown in Fig. 1b. Our method achieved good performance in Fig. 1c, achieving an average improvement of 13.0% RMSE and 24.5% MAPE, where the abscissa is the missing rates and the ordinate is the errors.

From Fig. 1, it is apparent that the Health-care dataset has mass missing values. When using and storing this dataset, if the "deletion" method [4] is employed, almost all the records in the dataset will be deleted. If the "mean" or "zero-value" imputation method [5] is utilized, the filled dataset will differ significantly from the original dataset. If the time series relation is exploited for imputation, it is impossible to establish an effective time series-based prediction model due to the mass missing values. If the GANs are utilized to learn the distribution of the original data, although the imputation accuracy is somewhat improved, it still does not reach the level of practical application.

Given those practical issues, in recent years, data with high missing rates imputation based on GAN network has been increasingly studied, where multivariate time series GAN network is a research hotspot. Early work attempted to employ GANs to learn the distribution patterns of multivariate electronic medical records [6, 7]. In recent years, methods combining multivariate time series data mining and GANs for missing value imputation have emerged. For example, Miao et al. [8] explored the time series classification method and the GANs model, and proposed a semi-supervised GAN imputation approach. Cao et al. [9] investigated the Recurrent Neural Networks (RNNs) model and proposed a time series data imputation method based on bidirectional RNN. Wang et al. introduced the attention mechanism [6] and proposed the STA-GAN model [10]. Based on these, Benchekroun et al. [11] studied the characteristics of heart rate variability physiological data with high missing rates, and applied several missing value imputation methods to fill these data.

Although proven to be effective, the uncertainty of GANs has not been considered, nor has the role of the GRUs (Gate Recurrent Units) based on time decay been explored in missing value imputation. This could potentially provide another method for electronic medical records imputation. This has motivated us to explore the utilization of time decay compensation and the UGAN (Uncertainty Generative Adversarial Network), which allows traditional GANs and GRUs to work together and form a new missing value imputation method, UGAN-GRUD (Uncertainty GAN-Gate Recurrent Unit Decay). In UGAN-GRUD, to overcome the challenge of capturing the distribution pattern of high-missing-rate datasets, we introduce the uncertainty matrix unit *U* into GAN to form the UGAN, which is an improvement not considered in existing methods. To utilize the time interval information and *U* in the dataset, we introduce the time decay factor ${v}_{t}$ into GRUs to form the GRUD (Gate Recurrent Unit Decay) network. We propose a dual-network collaborative training mechanism, where the uncertainty matrix *U* in GAN is outputted and utilized to guide the training of GRUD. Compared to the current state-of-the-art methods, our approach better captures the distribution of high-missing-rate datasets and performs more accurate imputation. Experimental results demonstrate that our method outperforms existing state-of-the-art methods.

In summary, the main contributions of this paper can be summarized as follows:

1. We propose the UGAN-GRUD model for the first time, where UGAN is employed to learn spatial distribution patterns and GRUD is leveraged to learn time series patterns. The combination of the two improves the imputation accuracy of high-missing-rate datasets.
2. We propose an improved method for GAN, which includes Generator *G*, Discriminator *D*, and uncertainty matrix *U*, called UGAN, which can capture the distribution patterns and uncertainty of the dataset more accurately. We also propose an improved method for GRU based on time decay factor, called GRUD, which can further improve the imputation accuracy.
3. We theoretically and experimentally demonstrate that the proposed UGAN-GRUD achieves better performance, and also discuss the impact of dataset dimensions on UGAN-GRUD.

The organization of the paper is as follows. Related work is introduced in Sect. 2. The proposed UGAN-GRUD model is detailed in Sect. 3, including the architecture of the model and the design of its components. In Sect. 4, experiments are conducted on three publicly available electronic medical record datasets, and the results are compared and analyzed. Section 5 concludes the study and discusses future research directions.

## Related work

In addition to the current state-of-the-art missing value imputation methods based on GANs, there are many other missing value imputation methods. In this section, we conduct a literature review of missing value imputation methods from four aspects: statistics, machine learning, deep learning, and electronic medical records.

### Imputation methods based on statistics

The imputation method based on statistics refers to filling the missing values with statistics method, such as the methods "constant ", "mean", and "sampling". For example, Park et al. [4] adopted the missing value imputation method based on "constant" in analyzing the sleep data. Robertson et al. [5] designed a missing value imputation method based on "mean". Further, Nickerson et al. [12] designed a missing value imputation method based on adjacent observations. Zhang et al. [13] modeled the probability distribution of data changes and used the probability distribution model to predict missing values. Later, Singh et al. [14] investigated statistical sampling and sample estimation, and proposed a method for filling missing values based on continuous "sampling". Therefore, the imputation methods based on statistics are suitable for discrete data imputation, and the imputation effect is better when the data follow a normal distribution.

### Imputation methods based on machine learning

Imputation methods based on machine learning include K-Nearest Neighbor (KNN) algorithm, shallow neural network method, and Matrix Factorization (MF) method, etc. For example, Ma et al. [15] proposed a missing values imputation method based on KNN clustering. Shallow neural network is an early form of neural network model, whose network structure is simple and the number of layers is small. Chen et al. [16] investigated the overfitting problem of neural networks, and proposed a neural network method based on steams. Tang et al. [17] employ a fuzzy neural network to classify the data, followed by the KNN method to predict the amount of missing values in each category, and finally utilize fuzzy rough sets to fill in missing values. The MF algorithm attempts to reconstruct the original data by matrix factorization to find the correlation between the data. In recent years, methods based on MF have been introduced into the time series data imputation. Generally, MF-based methods decompose a data matrix into two low-dimensional matrices, and then attempt to reconstruct the original matrix. During the process of matrix reconstruction, missing values are filled in. Fernandes et al. [18] proposed a MF-based method for filling missing values in multivariate time series data, and smoothed the filled values and observed values. Rios et al. [19] exploited machine learning methods for cardiovascular disease prediction and evaluated seven methods for filling in missing values. The imputation methods based on machine learning usually rely on prior knowledge of the data, which makes it difficult to deal with the potential rules in the data. In addition, most of the machine learning-based imputation methods emphasize the structure of data, so it cannot handle the unstructured data well.

### Imputation methods based on deep learning

The imputation methods based on deep learning exploit the powerful learning function of deep neural network to learn potential rules from the dataset, and then complete the prediction of missing values. RNNs can process time series data through iterative and scalable neurons, which can well remember the sequence relation of time series data, so as to effectively fill the missing values. Ouyang et al. [20] used RNN to learn the relation between data and time, and then utilized neural networks to predict missing values. Cao et al. [9] proposed a supervised learning-based time series imputation model named BRITS. BRITS assumes that all the labels of time series data are complete, and therefore, data without labels are discarded during the training. It is worth noting that in the datasets with high missing rates, BRITS usually results in severe overfitting due to the sharp reduction of training samples. Shukla et al. [21] improved the weights of BRITS and proposed the AUCOA model. GAN can generate new data from the distribution of the original data. Considering that the missing data and the non-missing data in the dataset follow the same distribution law, the data can be generated by the GAN to fill in the missing values. Yoon et al. [6] proposed a model GAIN that fills missing values through GAN. GAIN exploits the Generator to learn the distribution law of the original data with missing values, and leverages the Discriminator to judge the missing values produced by the Generator. Miao et al. [8] proposed a semi-supervised GAN model named SSGAN for missing values imputation. In SSGAN, a semi-supervised classifier is designed to iteratively classify unlabeled time series data and make the Generator produce predictions for missing values. The methods based on deep learning have greatly improved the accuracy of missing values imputation. However, for the datasets with high missing rates, the accuracy is still not high.

### Electronic medical record imputation

The problem of electronic medical record imputation is a clinical application-oriented issue that has gone through three stages of development. Early electronic medical record imputation employed traditional zero-value imputation methods [4], followed by the adoption of machine learning methods [19] for imputation. Currently, most electronic medical record imputation employs deep learning methods, such as the study by Zheng et al. [22] on predicting mortality risk, which utilized the LSTM-RUN model to fill missing values. Experimental results show that this method is effective, where LSTM is a special type of recurrent neural network. Shi et al. [23] applied GRU to the learning of clinical time series data and found that the GRU-based method is a fast missing value imputation method, with GRU being a simplified version of LSTM. The latest electronic medical record imputation methods are based on GAN networks, but GAN networks have only been applied in a simplistic manner [24, 25]. That is to say, these methods have not considered the specific needs of the electronic medical record field, and thus have not improved GAN networks accordingly. As a result, these methods have led to the problem of high missing rates not being adequately addressed.

To sum up, since the data are seriously missing, if the method "deletion" is utilized, almost all records in the dataset will be removed; if the method "mean" or "zero" is employed to fill, the filled dataset will be very different from the original dataset; if the time series method is exploited to fill, a time-based prediction model cannot be established due to the seriously data missing; if GANs are employed to fill, there will be a large deviation between the learned data distribution law and the real data distribution law. Therefore, the existing methods cannot effectively handle the serious problem of electronic medical record imputation with high missing rates.

## Imputation based on temporal GAN

An imputation method for electronic medical records based on GAN and temporal relation is proposed. The method first exploits GAN to learn the true distribution of the original data, and fill in the missing values with the generated data. Then, it leverages the time relation to rectify the filled values.

### Problem descriptions

1. High-dimensional electronic medical record: refers to electronic medical record that contain multiple medical features.

Let $x={\{x}_{0},{x}_{1},\dots ,{x}_{n-1}\}\in {\mathbb{R}}^{d\times n}$ denote electronic medical record dataset, where ${x}_{0}$ represents the observation value of ***x*** at time *t*_*0*_, and ${x}_{1}$ represents the observation value at time *t*_*1*_, and so forth. Each observation value includes *d* features, for example, ${x}_{0}^{j}$ represents the *j*^*th*^ feature value of ${x}_{0}$. In general, when *d* >  = 3, ***x*** is high-dimensional electronic medical record dataset.

(2) Missing value mask matrix: used to mark the missing status of high-dimensional electronic medical records.

Let $m({m}_{i}^{j})\in {\mathbb{R}}^{d\times n}$ mark the missing status of electronic medical record dataset ***x***, then,

1 $${{\varvec{m}}}_{{\varvec{i}}}^{{\varvec{j}}}=\left\{\begin{array}{cc}1& if\ {x}_{i}^{j} \ is \ observed\\ 0& if\ {x}_{i}^{j}\ is\ missing\end{array}\right.,$$

where ${{\varvec{m}}}_{{\varvec{i}}}^{{\varvec{j}}}$ is a flag in the mask matrix, and 0 means missing, 1 means normal.

(3) Missing interval matrix: used to mark time intervals.

Let ${\varvec{\delta}}({{\varvec{\delta}}}_{{\varvec{i}}}^{{\varvec{j}}})\in {\mathbb{R}}^{d\times n}$ denote the time interval matrix of electronic medical record dataset ***x***, then,

2 $${{\varvec{\delta}}}_{{\varvec{i}}}^{{\varvec{j}}}=\left\{\begin{array}{ll}0& if\ i==0\\ {t}_{i}-{t}_{i-1}& if\ {m}_{i-1}^{j}==1\ \&\&\ i>0,\\ {\delta }_{i-1}^{j}+{t}_{i}-{t}_{i-1}& if\ {m}_{i-1}^{j}==0\ \&\&\ i>0\end{array}\right.$$

where ${\varvec{\delta}}({{\varvec{\delta}}}_{{\varvec{i}}}^{{\varvec{j}}})$ is employed to compensate for time decay, and ${{\varvec{m}}}_{{\varvec{i}}-1}^{{\varvec{j}}}$ is the element in the mask matrix.${t}_{i}$ and ${t}_{i-1}$ are time.

The task of missing value imputation can be described as: based on the given dataset of high-dimensional electronic medical record ***x***, missing value mask matrix ***m***, and missing interval matrix ***δ***, establish a missing value imputation model, and predict the missing data.

### UGAN-GRUD model

**Fig. 2:**
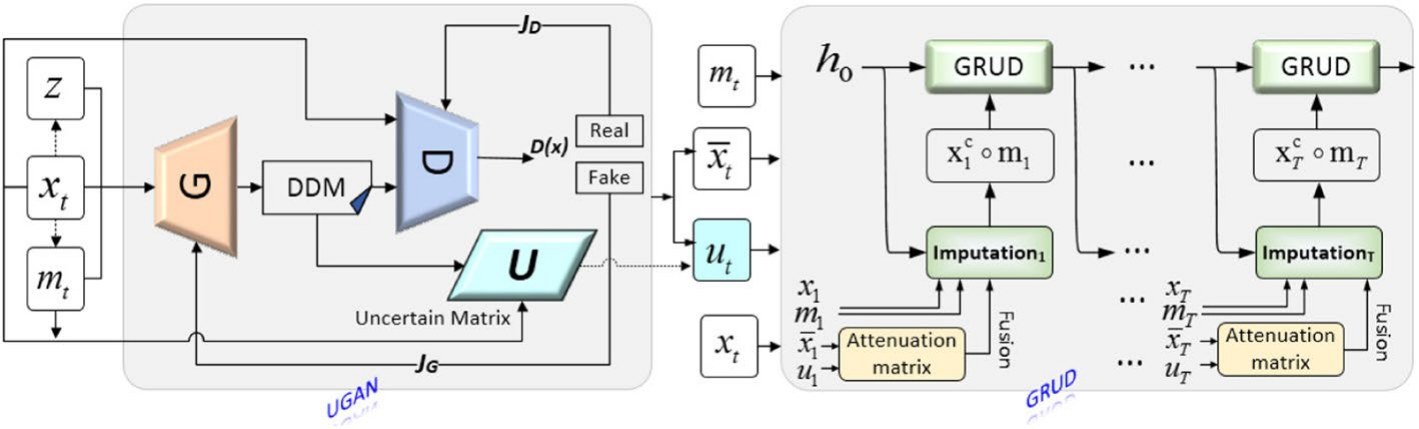
The illustration of model UGAN-GRUD. In Fig. 2, *G*, *D*, and *U* are the Generator, Discriminator, and uncertainty matrix, respectively. *z*, *x*_*t*_, and *m*_*t*_ are the inputs, where *z* is a random value, *x*_*t*_ is multivariate time series data with missing values, and *m*_*t*_ is the mask vector. *DDM* is the data distribution matrix produced by *G*; *D(x)* is the output of the *D*. *G* updates the neural network with its loss function *J*_*G*_; *D* updates the neural network with its loss function *J*_*D*_. ${m}_{t},{\overline{m} }_{t},{u}_{t},{and x}_{t}$ are inputs to the neural network GRUD for secondary imputation based on time decay compensation, where ${u}_{t}\in \text{U}$, and ${\overline{x} }_{t}\upepsilon DDM$. The neural network GRUD consists of *T* units, each corresponding to a specific set of ${m}_{t},{\overline{m} }_{t},{u}_{t},\text{and }{x}_{t}$

To overcome the imputation problem faced by high-missing-rate electronic medical records, we propose a missing value imputation model based on uncertainty matrix and time decay factor, viz., UGAN-GRUD. In the model UGAN-GRUD, in order to alleviate the problem of learning the distribution law of electronic medical records with high missing rates, we propose a control network UGAN based on the uncertainty matrix *U*, where *U* is the difference between the generated data and the original data, which represents the uncertainty of GAN. Due to the high missing rates of the original dataset, *U* changes drastically, and its values are uncertain. Considering the accuracy and diversity of imputation, we propose the GRUD based on time decay factor, where the time decay factor is an operator that uses time order and time interval to correct the filled data, which is a function of ${u}_{t}$ (${u}_{t}\in U$). The illustration of UGAN-GRUD is shown in Fig. 2.

In the proposed method, the potential distribution of electronic medical records is captured by the Generator, and the output data of the Generator are judged and optimized by the Discriminator. The Generator and the Discriminator form two opposing sides, so that they constantly optimize themselves and improve their ability to generate or discriminate. Eventually, the neural network becomes stronger during the training process. In the time decay compensation process on the right side of Fig. 2, we exploit the temporal dependencies between GRU units and the attenuation matrix to rectify the filled values of UGAN. Since the time intervals between missing values are not necessarily equal, it is necessary to obtain the information of time intervals. UGAN-GRUD not only considers the correlation of features and the uncertainty of GAN-generated data, but also exploits the correlation between time.

#### UGAN

The data generated by ordinary GANs is not accurate for filling in missing values in electronic medical records with high missing rates. To alleviate this issue, we propose an uncertainty matrix-based control network UGAN that takes into account the dynamics of the data distribution.

Unlike the ordinary GANs, UGAN consists of *G*, *D*, and *U*. The input of *G* is not only *z*, but a combination of *z*, *x*, and *m*, where *x* is the original input, *z* is a random matrix based on *x*, and *m* is a mask matrix based on *x*. In UGAN, to improve the optimization speed of the neural network, *tanh()* is selected as the activation function by *G* and *D*. The raw data are normalized, which are mapped between [-1.0, 1.0].

At a certain moment, the input of *G* is *x*_*t*_, *m*_*t*_, and *z*_*t*_, and the output is the data distribution matrix DDM, where DDM consists of a series of estimated values ${\widehat{x}}_{t}$.

3 $${\widehat{{\varvec{x}}}}_{{\varvec{t}}}={\varvec{G}}\left({{\varvec{x}}}_{{\varvec{t}}},{{\varvec{m}}}_{{\varvec{t}}},\left(1-{{\varvec{m}}}_{{\varvec{t}}}\right)\odot {{\varvec{z}}}_{{\varvec{t}}}\right),$$

where, $\odot$ is the element-wise multiplication and ${\widehat{x}}_{t}$ is the estimated value of the original input vector ${x}_{t}$. Regardless of whether there are missing values in ${x}_{t}$, *G* will generate the estimates in its corresponding dimensions, that is, the non-missing values in *x* have also corresponding estimates. It should be noted that zero is utilized as a placeholder for missing values in the dataset before the neural network is trained.

To rectify the values of the DDM, it is necessary to replace the corresponding values in the DDM with the non-missing values in ***x***, as shown in Eq. (4).

4 $${\overline{{\varvec{x}}} }_{{\varvec{t}}}={{\varvec{m}}}_{{\varvec{t}}}\odot {{\varvec{x}}}_{{\varvec{t}}}+\left(1-{{\varvec{m}}}_{{\varvec{t}}}\right)\odot {\widehat{{\varvec{x}}}}_{{\varvec{t}}},$$

where ${\overline{x} }_{t}$ is the corrected vector, *m*_*t*_ is the corresponding mask vector, ${x}_{t}$ is the corresponding original input vector, and ${\widehat{x}}_{t}$ is the output of Eq. (3). In order to measure the accuracy of the data generated by *G*, an uncertainty matrix *U* = $\{{u}_{1},{u}_{2}, ...,{u}_{t}\}$ is introduced, where *U* is the difference between the generated vector $\widehat{x}$ and the original data vector *x*, that is, at time *t*, the error between ${x}_{t}$ and ${\widehat{x}}_{t}$ can be calculated by Eq. (5).

5 $${{\varvec{u}}}_{{\varvec{t}}}=\sqrt{\frac{\sum_{i=1}^{d}{\left({x}_{t}^{i}\left(1-{m}_{t}^{i}\right)-{\widehat{x}}_{t}^{i}\left(1-{m}_{t}^{i}\right)\right)}^{2}}{k}}.$$

In Eq. (5), *d* is the dimension of multivariate time series data at time *t*, and *k* is the sum of the observations at time *t*. Since values in some dimensions at time *t* may be missing, $d\ge k$. ${u}_{t}$ represents the uncertainty of the filled data at time *t*, and it will be further exploited in subsequent neural networks GRUD.

*D* is responsible for judging the accuracy of the generated data. The main task of *D* is to calculate a probability value between 0 and 1 based on the true label, the original input, and the generated data. We make UGAN call the Discriminator twice, one for real data discrimination and the other for fake data discrimination. The different outputs of the Discriminator are leveraged to calculate the loss values of Generator and Discriminator. Finally, the parameters of the neural network are updated using the back-propagation mechanism. In Algorithm 1, UGAN is described in more detail.

**Figure:**
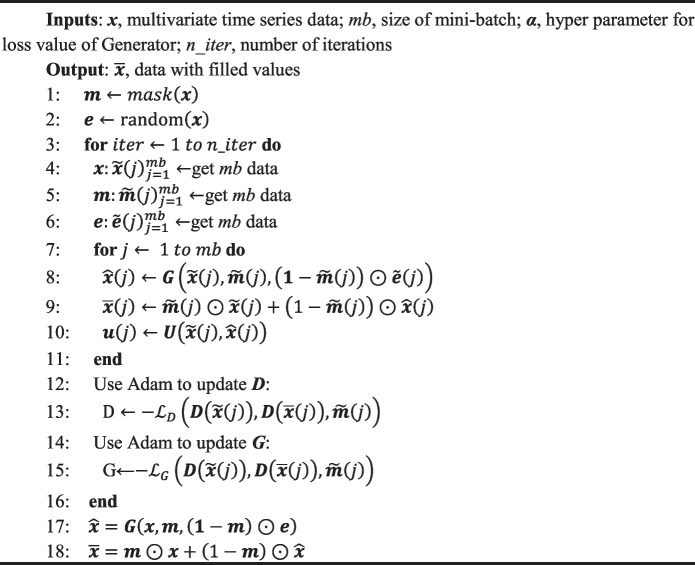
** Algorithm 1.** UGAN algorithm

During the training of UGAN, samples need to be extracted from the training dataset, and these samples will be utilized to generate the mini-batches used in the iterations, denoted as $\widetilde{x}$, $\widetilde{m}$ and $\widetilde{e}$. Briefly, the main steps of the algorithm UGAN are as follows.

1. Take the samples $\widetilde{x}$, $\widetilde{m}$, and $\widetilde{e}$;
2. Generate the data $\widetilde{x}$ according to Eq. (3);
3. Calculate the loss function of ***D*** using Eq. (6);

6 $${\mathcal{L}}_{G}={\nabla }_{{\theta }_{D}}\frac{1}{mb}\sum\nolimits_{j=1}^{mb}\left[\widetilde{{\varvec{m}}}\left(j\right)\odot D\left(\widetilde{{\varvec{x}}}\left(j\right)\right)\right]+\frac{1}{mb}\sum\nolimits_{\text{j}=1}^{\text{mb}}\left[\left(1-\widetilde{{\varvec{m}}}\left(j\right)\right)\odot {\varvec{D}}\left(\overline{{\varvec{x}} }\left(j\right)\right)\right].$$

(4) Calculate the loss function of ***G*** using Eq. (7)

7 $${\mathcal{L}}_{D}={\nabla }_{{\theta }_{G}}\frac{1}{mb}\sum\nolimits_{j=1}^{mb}\left[\left(1-\widetilde{{\varvec{m}}}\left(j\right)\right)\odot D\left(\overline{{\varvec{x}} }\left(j\right)\right)\right]-\frac{\alpha}{mb}\sum\nolimits_{j=1}^{mb}\left[\widetilde{{\varvec{m}}}\left(j\right)\odot {\left(\widetilde{{\varvec{x}}}\left(j\right)-\overline{{\varvec{x}} }\left(j\right)\right)}^{2}\right].$$

(5) Repeat the training within a given number of iterations (*n_iter*);
(6) Obtain the electronic medical record dataset with filled values after training UGAN.

#### GRUD

In order to further rectify the missing values filled by UGAN, we propose an iterative and scalable neural network structure GRUD. In GRUD, by introducing a time decay factor, the missing data will be filled differently according to the time of its missing, which increases the diversity of missing value imputation. GRUD provides corresponding information by memorizing the sequential relationship and historical time information of time series data.

**Fig. 3:**
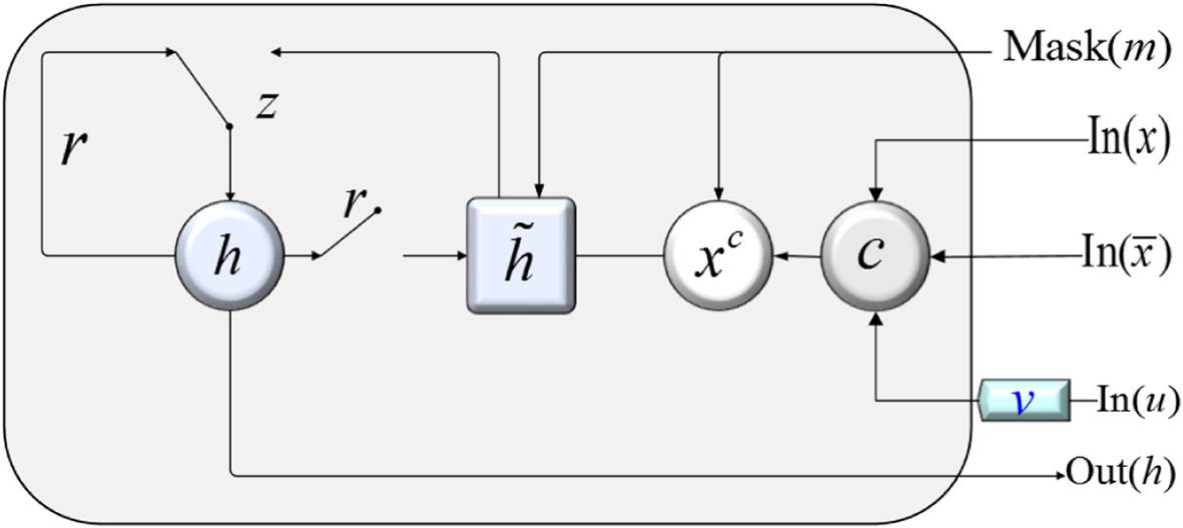
GRUD based on time decay factor. In Fig. 3, ***z*** represents the update gate, *r* represents the reset gate, $\widetilde{h}$ represents the candidate hidden state, ${x}^{c}$ represents the complete vector, *c* represents the estimated value, and *v* represents the time decay factor. *Mask*(*m*), *In*(*x*), *In*($\overline{x }$), *In*(*u*) represent the four inputs, and *Out*(*h*) represent the output

For electronic medical records, the issue of missing for a long time often arises [24, 25]. For the long-term missing of electronic medical records, we attenuate the historical memory vector according to the length of the missing time: if the missing time is long, due to the principle of forgetting, the historical information has little influence on the current status, so the historical memory vector should be attenuated greatly; otherwise, if the missing time is short, the historical memory vector should undergo a small decay. In order to adapt to the missing time intervals of electronic medical records, we propose the GRUD based on a time decay factor, as shown in Fig. 3.

The time decay matrix is composed of time decay factors, which exploits the sequential and historical information between time that can finely fill in the missing data. Specifically, the time decay matrix $V({v}_{t})$ is calculated by Eq. (8).

8 $${{\varvec{v}}}_{{\varvec{t}}}=\text{exp}\left\{-\text{max}\left(0,{{\varvec{W}}}_{u}{{\varvec{u}}}_{t}+{{\varvec{b}}}_{u}\right)\right\},$$

where ${W}_{u}$ is the weight parameter, ${b}_{u}$ is the bias vector, ${u}_{t}$ is the error between ${x}_{t}$ and ${\widehat{x}}_{t}$ at time *t*, and the range of ${v}_{t}$ is [0, 1]. ${u}_{t}$ is exploited in ${v}_{t}$. ${u}_{t}$ is the deviation between the vector generated by UGAN and the original data vector, which can be employed to further improve the diversity and accuracy of the filled values. Therefore, ${u}_{t}$ is introduced into the GRUD to further fill in the missing data by using the time associations.

Obviously, ${v}_{t}$ is leveraged to highlight the reliability of the generated imputation values, which can rectify the attention of the large biased data generated by *G*. The estimated value ${x}_{t}^{r}$ of the current sequence can be predicted from the hidden layer state ${\widehat{h}}_{t-1}$.

9 $${{\varvec{x}}}_{{\varvec{t}}}^{{\varvec{r}}}={{\varvec{W}}}_{r}{\widehat{{\varvec{h}}}}_{t-1}+{{\varvec{b}}}_{r}.$$

Based on ${v}_{t}$, ${x}_{t}^{r}$ and ${\overline{x} }_{t}$ are combined to obtain the estimated value of GRUD, as shown in Eq. (10).

10 $${{\varvec{c}}}_{{\varvec{t}}}={{\varvec{v}}}_{{\varvec{t}}}{\overline{{\varvec{x}}} }_{t}+{\left(1-{{\varvec{v}}}_{{\varvec{t}}}\right){\varvec{x}}}_{t}^{r}.$$

Finally, replace the missing values with the estimated values ${c}_{t}$ to get the complete vector ${x}_{t}^{c}$, as shown in Eq. (11).

11 $${{\varvec{x}}}_{{\varvec{t}}}^{{\varvec{c}}}={{\varvec{m}}}_{{\varvec{t}}} \odot {{\varvec{x}}}_{{\varvec{t}}}+(1-{{\varvec{m}}}_{{\varvec{t}}}) \odot {{\varvec{c}}}_{{\varvec{t}}}.$$

Additionally, the "∘" operator needs to be leveraged to concatenate the complete vector with the corresponding mask vector. For the hidden state ${h}_{t-1}$, ${v}_{t-1}$ is employed for processing to get ${\widehat{h}}_{t-1}$. Therefore, the update of hidden state at time *t*, ${h}_{t}$, is shown in Eq. (12).

12 $${{\varvec{h}}}_{t}=\sigma \left({{\varvec{W}}}_{h}{\widehat{{\varvec{h}}}}_{{\varvec{t}}-1}+{{\varvec{P}}}_{h}\left[{{\varvec{x}}}_{t}^{c}\circ {{\varvec{m}}}_{t}\right]+{{\varvec{b}}}_{h}\right),$$

where, $\sigma$ represents the activation function, ${W}_{h}$ and ${P}_{h}$ are the weight parameters, and ${b}_{h}$ is the bias vector. The specific definition of the loss function is shown in Eq. (13).

13 $$\mathcal{L}=\sum\nolimits_{n=1}^{N}\sum\nolimits_{t=1}^{T}{\mathcal{L}}_{MAE}({{\varvec{x}}}_{t}^{(n)}\odot {{\varvec{m}}}_{t}^{(n)},{{\varvec{c}}}_{t}^{(n)}\odot {{\varvec{m}}}_{t}^{(n)}).$$

In Eq. (13), ${\mathcal{L}}_{MAE}$ denotes the mean absolute error loss, and the meanings of ${x}_{t}$, ${m}_{t}$, and ${c}_{t}$ are the same as those described above. Algorithm 2 describes the entire procedure of GRUD in detail.

**Figure:**
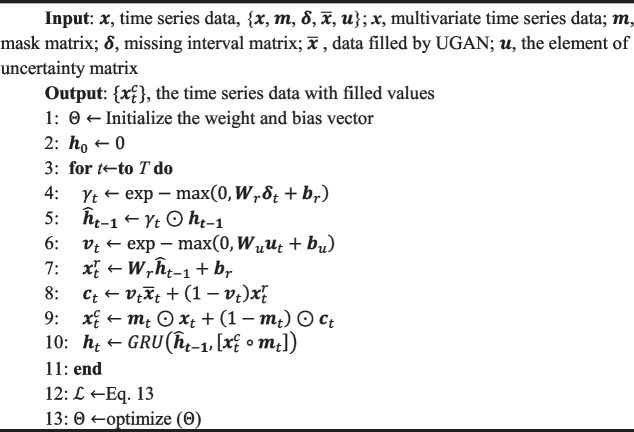
** Algorithm 2.** GRUD Algorithm

The model UGAN-GRUD includes two parts, one is the deep neural network UGAN, and the other is the deep neural network GRUD. The former learns the distribution law of the original dataset through the Generator, guides the Generator through the Discriminator, and records the deviation of the filled values through the uncertainty matrix. The latter exploits GRUD to memorize the sequence relations and historical time information of time series data, and then employs the learning function of deep neural network to discover the correlations between data. Finally, the target of improving the imputation accuracy for the datasets with high missing rates is achieved.

## Experiments and analysis

To validate the model UGAN-GRUD, we conducted three aspects of experimental studies: (1) the performance study, (2) the ablation study, and (3) the efficiency study. Like existing methods [4, 5, 8, 10, 12, 21], we performed the same dataset selection and experimental parameter settings.

### Experimental datasets and baseline models

To verify the effectiveness of the UGAN-GRUD model, three publicly available e-health datasets, Health-care [3], Perf-DS1 [26–28] and Perf-DS2 [28] were used. Those electronic medical records are the data on human physiological indicators [3, 28]. The datasets are provided by the intensive care units and community hospitals, and the indicators involved include body temperature, heart rate, blood sugar content, electrocardiogram, and so forth. The Health-care dataset has a total of 4,000 records, each 24–36 h long, and belongs to multivariate time series data. Most of the records of Health-care dataset are incomplete (components missing), it has an average missing rate of 80.67%, and the related main task is to classify patients. The Perf-DS1 dataset has a total of 90,000 records, and its average missing rate is 50%, whose continuous missing problem is serious. The Perf-DS2 dataset has a total of 12,000 records, and its average missing rate is 13%, and there is obvious periodicity in these data.

According to the experiments of the current state-of-the-art methods [8, 21], the division ratio of the training dataset and the test dataset is 7:3, and they are used for training and testing, respectively. Since the missing-value-imputation based on traditional statistical methods does not require training, it directly enters the testing phase. In order to simulate the mass missing phenomenon, secondary missing processing is required. The method of secondary missing processing [6] is to randomly select a record, and if it is a complete record, delete it and mark it as missing data; and if it is a record with missing values, select next record to handle. We employ a normal distribution with a random seed of 1024 to randomly select the serial number/position of the record in the dataset.

In the research, models such as Zero [4], Mean [5], Last [12], KNN [29], STA-GAN [10], AUCOA [21], SSGAN [8] were selected as the baseline methods for comparisons.

- ∎ Zero[4] model: This is a classic model that features the use of 0 to fill in missing values.
- ∎ Mean [5] model: This is also a widely used classic model, characterized by using the global average to fill in missing values.
- ∎ Last [12] model: This is a widely used model in the field of behavioral data mining, which features the use of the last observations to fill in the missing values.
- ∎ KNN [29] model: It is also called the K-Nearest Neighbor imputation algorithm, which is characterized by using the KNN algorithm to find the samples with "near neighbor", and then employing the weighted average of the "near neighbor" samples to fill in missing values.
- ∎ STA-GAN model [10]: This is a missing value imputation model based on GAN network, which fills missing values through the Hint Matrix mechanism [18].
- ∎ AUCOA [21] model: This is a time-series neural network model that is characterized by bidirectional training of data. One direction arranges the data and trains them along time increments, and the other direction arranges the data and trains them in decreasing time. Experiments showed that this bidirectional training method can improve the accuracy of missing value imputation of time-series data. SSGAN [8] model: This is an improved GAN network model that is characterized by iteratively classifying unlabeled time series data through a semi-supervised classifier, which in turn assists the Generator to estimate missing values by using these classified data.
- ∎ SSGAN [8] model: This is an improved GAN network model that is characterized by iteratively classifying unlabeled time series data through a semi-supervised classifier, which in turn assists the Generator to estimate missing values by using these classified data.
- **∎ UGAN-GRUD **model: The method proposed in this paper.

Since the problem solved by some baseline methods is the missing value imputation for the general purpose domain, and the problem we are solving is the missing value imputation for the biomedical field, we utilize the datasets of the biomedical field [3, 28] to re-compare these methods. In the experiments, based on the characteristics of the datasets, we utilized a normal distribution to initialize the parameters in the models. In addition, as in Ref. [8–10], the neural network models were set a Batch Size of 128 and an Iterative Period (epoch) of 1000; The Adam optimizer was chosen for stochastic gradient descent training with a learning rate of 0.001, and the Sigmoid was chosen as the activation function to map variables between 0 and 1. To prevent the distribution of the dataset from adversely affecting the training process, all data were normalized so that their means were zero.

### Evaluation criteria

To facilitate evaluation and comparison, the Root Mean Squared Error (RMSE) [30] and the Mean Absolute Percentage Error (MAPE) [31] between the ground-truth values and the predicted values, are adopted as the evaluation criteria in this paper, as shown in Eqs. (14) and (15).

14 $$\mathrm{RMSE}=\sqrt{\frac1n{\sum\nolimits_{1=1}^n}{\left(y_1-y_i^{\prime}\right)^{2}}},$$

15 $$\text{MAPE}=\frac{100\%}{n}\sum\nolimits_{i=1}^{n}|\frac{{y}_{i}-{y}_{i}^{\prime}}{{y}_{i}}|.$$

In Eq. (14) and (15), *n* represents the number of samples, and ${y}_{i}$ and ${y}_{i}^{\prime}$ denote the ground-truth value and predicted value at time *i*, respectively. RMSE and MAPE represent the gap between the original data and the filled data. The smaller the RMSE and MAPE, the better the performance.

To evaluate the classification effect of the filled data, the Area Under Curve (AUC) metric is adopted in this paper, as shown in Eq. (16). The metric AUC represents the area under the Receiver Operating Characteristic (ROC) curve. The metric AUC is not sensitive to the proportion of positive and negative samples, so the metric AUC can better distinguish the pros and cons of the binary classification models [9].

16 $$\text{AUC}=\frac{1}{\left|{D}^{+}\right|+|{D}^{-}|}=\sum_{{x}^{+}\in {D}^{+}}\sum_{{x}^{-}\in {D}^{-}}f({x}^{+})>f({x}^{-}),$$

where ${D}^{+}$ represents the set of all positive samples, ${D}^{-}$ represents the set of all negative samples, and $f({x}^{+})>f({x}^{-})$ indicates that the prediction result of positive sample ${x}^{+}$ is better than that of negative sample ${x}^{-}$.

### Performance study

#### Performance of imputation

In the experiments, we implemented all the evaluation testbeds using PyTorch. To evaluate the missing values imputation performance of UGAN-GRUD, it is necessary to select homogeneous and comparable methods. In this paper, Zero, Mean, Last, KNN, STA-GAN, AUCOA, SSGAN were selected as comparison methods. At the same time, in order to reflect the processing effect of the high-missing-rate datasets, the datasets Health-care, Perf-DS1 and Perf-DS2 were treated with secondary missing, and the missing positions of records were randomly selected according to the normal distribution. As references [6, 8, 9], the "underscore" identification method was introduced to mark the top three models that performed better, and "bold" was used to mark the model that performed best in the experiments. Table 1 shows the imputation performance of different models on the dataset Perf-DS1 with different missing rates.

**Table 1:** Imputation performance experiments on the dataset perf-DS1. In Table 1, "Criteria" denotes evaluation criteria, which include RMSE and MAPE. Zero, Mean, Last, KNN, STA-GAN, AUCOA, SSGAN, and UGAN-GRUD are eight models used to compare

Models	Criteria	Missing rates							
		10%	20%	30%	40%	50%	60%	70%	80%
Zero 2019 [4]	RMSE	57.58	57.60	57.59	57.57	57.59	57.59	57.59	57.60
	MAPE	100.0%	100.0%	100.0%	100.0%	100.0%	100.0%	100.0%	100.0%
Mean 2017 [5]	RMSE	10.00	12.14	12.25	12.23	12.16	12.16	12.17	12.19
	MAPE	23.71%	31.00%	31.79%	31.08%	31.00%	30.79%	30.89%	31.07%
Last 2018 [12]	RMSE	7.63	7.96	8.30	8.76	9.31	10.04	11.06	12.57
	MAPE	11.65%	12.20%	12.91%	13.52%	14.38%	15.60%	17.28%	19.97%
KNN 2022 [29]	RMSE	5.02	5.19	5.33	5.50	5.73	6.02	6.44	7.15
	MAPE	9.60%	9.98%	10.42%	10.90%	11.51%	12.29%	13.59%	15.87%
STA-GAN 2023 [10]	RMSE	7.26	9.34	13.18	20.79	26.72	35.91	43.08	43.49
	MAPE	13.17%	17.21%	23.18%	34.03%	44.75%	59.19%	69.89%	70.60%
AUCOA 2023 [21]	RMSE	5.36	5.72	6.56	6.93	7.32	7.74	8.45	9.88
	MAPE	10.23%	10.87%	11.72%	12.15%	13.34%	14.23%	17.83%	18.33%
SSGAN 2021 [8]	RMSE	6.42	6.51	6.61	7.04	7.41	8.02	8.57	10.3
	MAPE	10.44%	11.04%	12.70%	13.02%	13.73%	14.80%	18.52%	18.94%
**UGAN-GRUD**	RMSE	**4.09**	**4.21**	**4.39**	**4.45**	**4.62**	**4.83**	**5.09**	**5.32**
	MAPE	**6.71%**	**7.02%**	**7.34%**	**7.61%**	**8.05%**	**8.62%**	**10.01%**	**10.56%**

The underline indicates the top-3 performance, while the bold indicates the best performance

It is easy to see from Table 1 that the UGAN-GRUD model achieves the best performance. Compared with the model Zero, the performance of UGAN-GRUD is greatly improved by 50%. The model AUCOA has the second performance, but its performance of imputation drops drastically as the missing rate increases. UGAN-GRUD has an average improvement of 36.2% in RMSE and 39.4% in MAPE compared to AUCOA. UGAN-GRUD has an average improvement of 39.2% in RMSE and 41.8% in MAPE compared to SSGAN. Table 2 shows the imputation performance of different models on the dataset Perf-DS2 with different missing rates.

**Table 2:** Imputation performance comparisons on the dataset perf-DS2. In Table 2, the evaluation criteria and missing rates are the same as those in Table 1

Models	Criteria	10%	20%	30%	40%	50%	60%
Zero 2019 [4]	RMSE	48.54	49.08	49.50	49.39	49.23	49.37
	MAPE	100.0%	100.0%	100.0%	100.0%	100.0%	100.0%
Mean 2017 [5]	RMSE	36.52	36.94	37.57	37.65	37.72	38.14
	MAPE	1107%	1089%	1102%	1097%	1095%	1066%
Last 2018 [12]	RMSE	12.66	13.96	15.67	16.83	17.87	19.54
	MAPE	27.66%	31.03%	33.29%	36.89%	41.42%	47.59%
KNN 2022 [29]	RMSE	14.33	15.28	17.02	18.97	22.40	27.21
	MAPE	37.03%	40.06%	44.30%	53.32%	70.96%	104.2%
STA-GAN 2023 [10]	RMSE	13.16	18.42	25.46	29.96	38.46	65.60
	MAPE	12.23%	29.48%	38.49%	49.68%	60.57%	84.98%
AUCOA 2023 [21]	RMSE	10.57	11.03	14.43	15.50	16.72	18.56
	MAPE	16.55%	**27.49%**	**32.01%**	**35.75%**	**40.31%**	45.61%
SSGAN 2021 [8]	RMSE	11.97	11.81	14.62	15.99	17.14	18.78
	MAPE	12.63%	28.95%	34.76%	39.90%	47.27%	50.22%
**UGAN-GRUD**	RMSE	**8.22**	**8.94**	**12.13**	**12.79**	**13.67**	**13.95**
	MAPE	**11.67%**	28.55%	35.15%	42.51%	57.47%	**43.96%**

The underline indicates the top-3 performance, while the bold indicates the best performance

It is easy to see from Table 2 that UGAN-GRUD performs the best on the criteria RMSE. From an average performance perspective, UGAN-GRUD has an average improvement of 19.7% compared to AUCOA and 22.8% compared to SSGAN. In terms of MAPE indicators, UGAN-GRUD is slightly lower than AUCOA, because (1) the periodicity of the Perf-DS2 dataset is better, and it is likely that UGAN and GRUD destroy the original time series laws of the data; (2) The initial missing rate of the Perf-DS2 dataset is relatively low, which makes the advantages of the UGAN-GRUD method impossible to play to a certain extent. This indicates that the UGAN-GRUD method is more suitable for datasets with random distribution and high missing rates. Table 3 shows the imputation performance of different models on the dataset Health-care with different missing rates.

**Table 3:** Imputation performance comparisons on the dataset health-care

Models	10%		20%	
	RMSE	MAPE	RMSE	MAPE
Zero 2019 [4]	144.16	100%	156.02	100%
Mean 2017 [5]	136.35	330.69%	149.97	311.56%
Last 2018 [12]	121.95	45.04%	135.55	46.04%
KNN 2022 [29]	162.44	46.71%	140.18	42.12%
STA-GAN 2023 [10]	34.46	5.92%	52.03	11.02%
AUCOA 2023 [21]	37.17	11.14%	53.88	12.67%
SSGAN 2021 [8]	32.32	8.31%	51.09	10.89%
**UGAN-GRUD**	**31.75**	**5.35%**	**48.09**	**10.21%**

The underline indicates the top-3 performance, while the bold indicates the best performance

It can be seen from Table 3 that the UGAN-GRUD model can still achieve better performance under the condition of large data loss, where the initial missing rate of the Health-care dataset is 80.67%. Other models that achieved better performance were STA-GAN and SSGAN, with SSGAN in second place and STA-GAN in third. UGAN-GRUD improved RMSE by an average of 7.7% and MAPE by 8.1% over STA-GAN model. UGAN-GRUD improved RMSE by an average of 4.3% and MAPE by 19.0% over SSGAN model.

Analysis: From the performance experiments of imputation, it can be seen that the UGAN-GRUD model performs well on the datasets Health-care, Perf-DS1, and Perf-DS2. It should be noted that the comprehensive missing rates of the datasets Health-care and Perf-DS1 are relatively high, while the comprehensive missing rate of the dataset Perf-DS2 is relative low. This indicates that the UGAN-GRUD model is not only a method suitable for high-missing-rate datasets, but also has certain reference value for common missing rate datasets.

#### Performance of classification and regression

Since the ultimate purpose of electronic medical record imputation is to support decision-making, the performance of classification and regression of the filled data need to be evaluated. Like references [6, 8, 9], we constructed a RNN classifier and a RNN regression predictor, and trained the models using the filled dataset. The number of training iterations is 30, the learning rate is 0.005, the dropout is 0.5, and the dimension of the hidden state in the RNN is 64. The evaluation criteria used for the classification is AUC, and the evaluation criteria used for the regression prediction is RMSE.

**Fig. 4:**
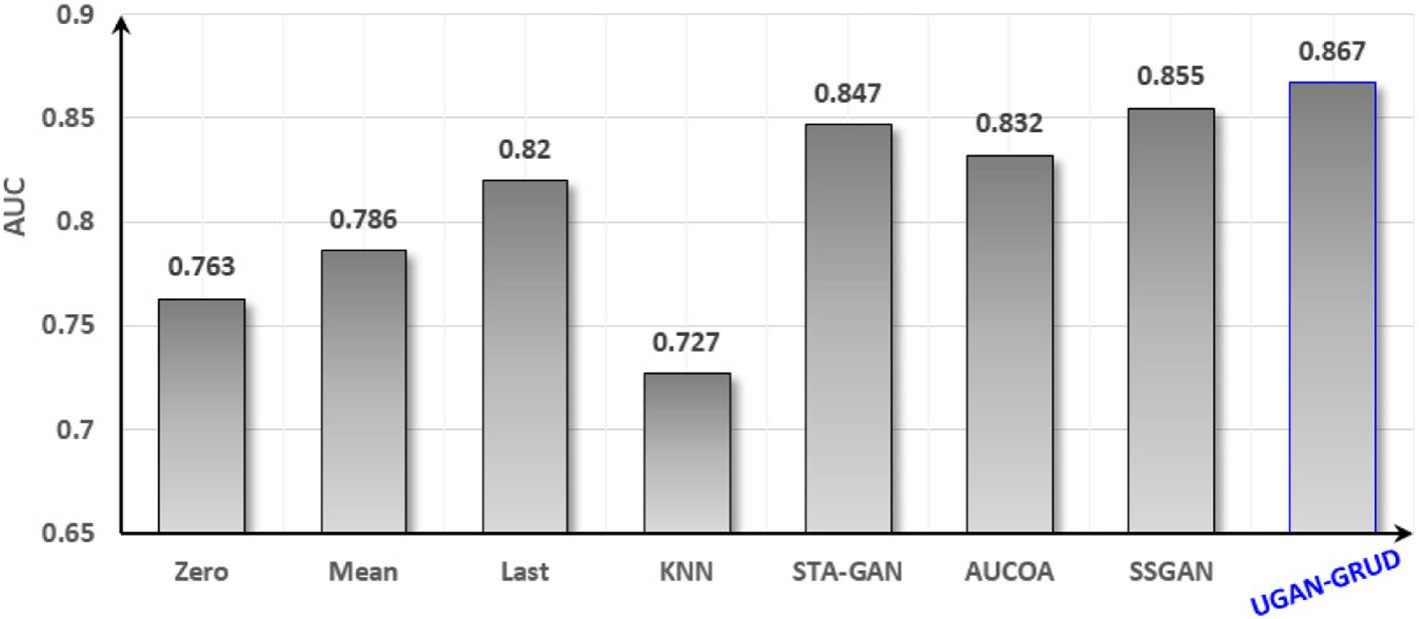
Classification performance of Health-care after filled. In Fig. 4, the abscissa is the eight compared methods, i.e., Zero, Mean, Last, KNN, STA-GAN, AUCOA, SSGAN, and UGAN-GRUD, and the ordinate is the AUC evaluation criterion

The Health-care dataset was used for the training and testing process of the classification task with a number of 30 classes. The Perf-DS1 dataset and the Perf-DS2 dataset were used for the training and testing process of the regression task. Figure 4 is the classification performance based on the Health-care dataset.

**Fig. 5:**
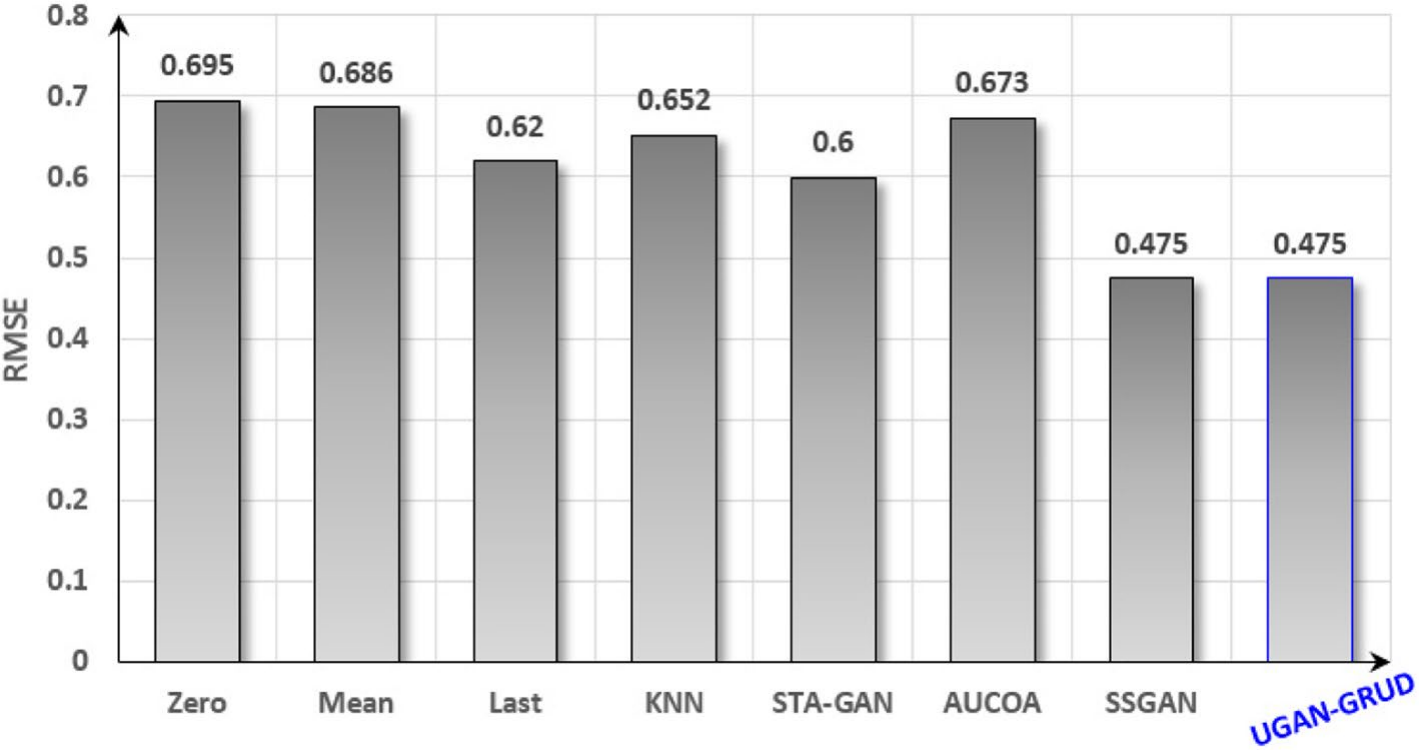
Regression performance of Perf-DS1 after filled. In Fig. 5, the abscissa is the eight compared methods, i.e., Zero, Mean, Last, KNN, STA-GAN, AUCOA, SSGAN, and UGAN-GRUD, and the ordinate is the evaluation criterion RMSE

It is easy to see from Fig. 4 that the classification performance is the best after the dataset is filled in by the method UGAN-GRUD, which is 19.2% higher than KNN method and 1.4% higher than SSGAN method. Figure 5 shows the regression task performed on the filled Perf-DS1 dataset, where the smaller the RMSE, the better the regression effect.

**Fig. 6:**
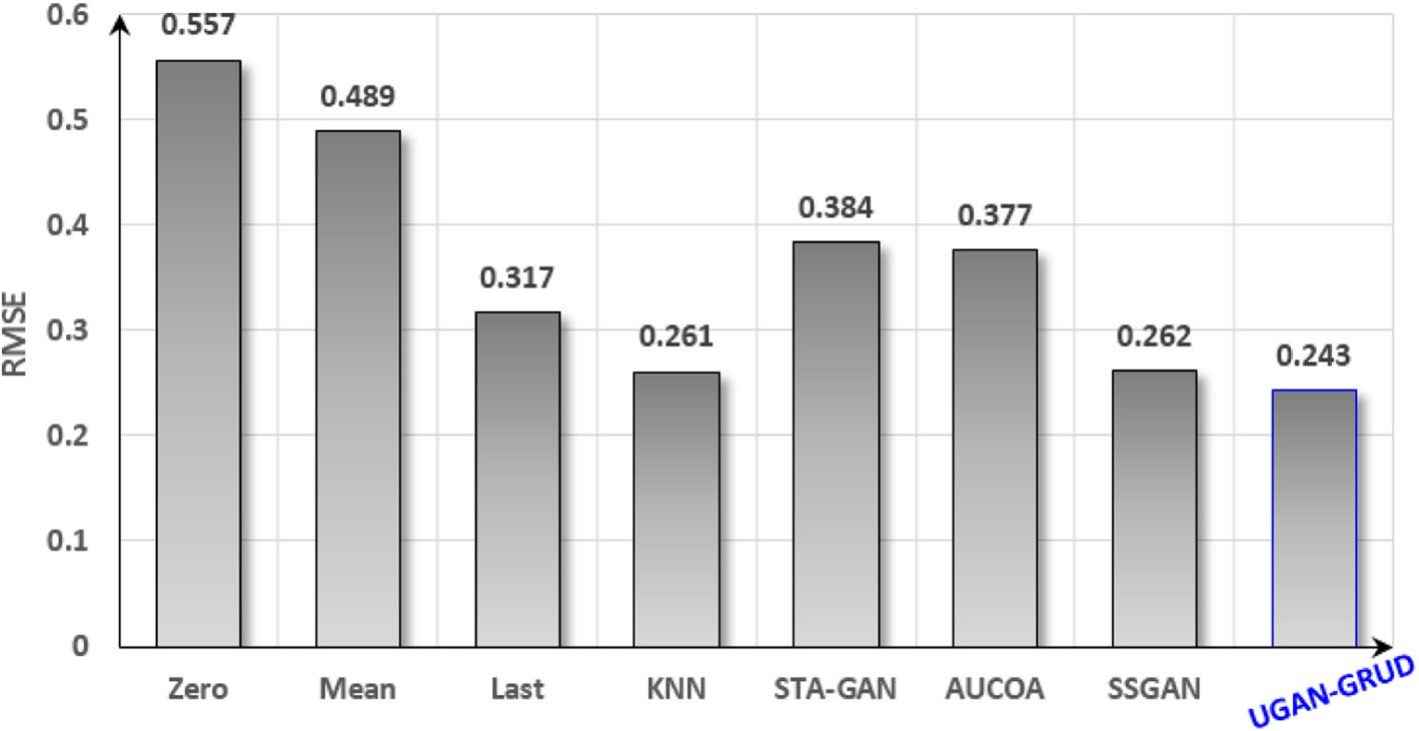
Regression performance of Perf-DS2 after filled. The meanings of the abscissa and the ordinate are the same as those of Fig. 5

Figure 6 shows the effect of performing regression tasks on the filled Perf-DS2 dataset, where the smaller the RMSE, the better the regression effect.

It is easy to see from Fig. 5 and Fig. 6 that the regression effect is different after the dataset is filled by different methods, among which UGAN-GRUD corresponds to the best effect, followed by SSGAN, and STA-GAN third. Classification and regression effects are inseparable from imputation effects, for example, UGAN-GRUD, SSGAN, AUCOA, STA-GAN methods with better imputation effects, and their corresponding classification and regression effects are also better. The imputation of dataset is a meaningful endeavor.

### Ablation study

#### Ablation experiments

To explore the impact of various improvements in the UGAN-GRUD model on performance, an ablation study is required. This means removing the improved parts in the UGAN-GRUD model and observing changes in model performance. The key improvements in the UGAN-GRUD model are two-fold, namely UGAN and GRUD. We utilized a GAN-based model [6] as the "Base" model. Then, we added GRUD to the "Base", called "Base + GRUD"; and we added UGAN to the "Base", called "Base + UGAN". Finally, we added both of these key improvements together, called "Base + GRUD + UGAN", which is also the UGAN-GRUD model. All neural network parameters were initialized with the same values. Table 4 shows the ablation study results of the UGAN-GRUD model.

**Table 4:** Ablation experiments of UGAN-GRUD model. In Table 4, Perf-DS1, Perf-DS2, and health-care are three datasets

Datasets	*Base*		*Base* + *GRUD*		*Base* + *UGAN*		**UGAN-GRUD**	
	RMSE	MAPE	RMSE	MAPE	RMSE	MAPE	RMSE	MAPE
Perf-DS1	12.4	19.5%	10.3	16.22%	7.2	13.1%	**4.09**	**6.71%**
Perf-DS2	14.5	23.9%	14.34	17.51%	13.2	12.2%	**8.22**	**11.67%**
Health-care	86.2	17.6%	55.3	11.3%	34.5	5.9%	**31.75**	**5.35%**

### Analysis

1. From Table 4, it can be seen that after adding GRUD to the "Base", the model's performance is improved. Since GRUD can mine the correlation from time series data, this indicates that GRUD helps to improve the accuracy of imputation. Similarly, after adding UGAN to the "Base", the model's performance is significantly improved. This shows that using an uncertainty matrix to capture the distribution of high missing rate datasets is an effective method.
2. Additionally, when both GRUD and UGAN are added to the "Base", the model's performance reaches its optimum. This indicates that the key improvements GRUD and UGAN are not only effective individually but also when combined, the overall performance can reach its best. In summary, the improvements of the UGAN-GRUD model are all effective, making it a competitive model.

#### Data dimension study

**Fig. 7:**
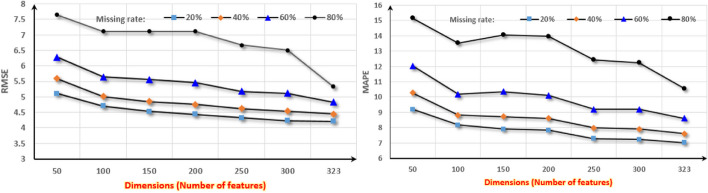
Impacts of different data dimensions on UGAN-GRUD. In Fig. 7, the abscissa is the data dimensions. The ordinate of the left graph is RMSE, and the ordinate of the right graph is MAPE

The impact of data dimension on the model refers to the impact of the number of features included in the dataset on the model. To explore the impact of data dimension on the model UGAN-GRUD, it is necessary to select a dataset with a larger number of features in the original dataset. Due to the large number of features in the dataset Perf-DS1, the Perf-DS1 was selected to verify the impact of the data dimension on UGAN-GRUD. RMSE and MAPE are still chosen as the evaluation criteria. Figure 7 is the experiments of the impacts of data dimension on the model UGAN-GRUD.

Analysis: From Fig. 7, it is easy to see, the two evaluation criteria RMSE and MAPE show relatively stable trends with the change of data dimensions. Since the RMSE and MAPE of the model UGAN-GRUD do not change significantly with the changes of the data dimensions, that shows the data dimension has less impact on the model UGAN-GRUD. In addition, different missing rates have a certain impact on performance. For example, when the missing rate is greater than 80%, the data dimension will have an impact on the model UGAN-GRUD as shown in Fig. 7.

### Efficiency study

In order to evaluate the efficiency of model training, we compared the training time of the four models: STA-GAN, AUCOA, SSGAN, and UGAN-GRUD. Table 5 shows the training time of the four models of STA-GAN, AUCOA, SSGAN, and UGAN-GRUD on the three datasets of Health-care, Perf-DS1, and Perf-DS2.

**Table 5:** Training Time of Models. In Table 5, STA-GAN, AUCOA, SSGAN, UGAN-GRUD are the four compared models. Perf-DS1, Perf-DS2, Health-care are three datasets. The data in the table represent the training time in seconds

Models	Perf-DS1	Perf-DS2	Health-care
STA-GAN [10]	8.746 s	13.43 s	5.84 s
AUCOA [21]	344 s	429.2 s	434 s
SSGAN [8]	376 s	478 s	480 s
**UGAN-GRUD**	147 s	154 s	162 s

It is easy to see from Table 5 that the training efficiency of the GAN series of imputation models is high, among which the STA-GAN model has the highest training efficiency but the worst performance, and the training efficiency of other improved models is reduced to varying degrees. The performance of the UGAN-GRUD model is the first and the training efficiency is second. Since the UGAN-GRUD model increases the computation of the uncertainty matrix and the training of the GRU neural network, it is not as efficient as the STA-GAN model. However, the performance of the UGAN-GRUD model far exceeds that of the STA-GAN model. Considering both performance and efficiency, the UGAN-GRUD model is the best choice.

### Discussion on scalability and limitations

Missing value imputation is used to restore data in real-world domains and plays an important role in intelligent decision-making. Although the method proposed in this paper is limited by the characteristics of electronic medical records research, it can be tried in scenarios with high missing rates. For example, in our experiments, we have attempted to employ the method proposed in this paper to process the datasets involved in references [8, 10, 21], etc., and the experimental results show that their performance has been improved to some extent. Since the research task of this paper is the missing value imputation of electronic medical records, no further research and experimental comparisons have been conducted on this. This will be one of the contents of our future research.

## Conclusion

The missing of electronic medical records is a commonly observed phenomenon that holds significant research value. In this paper, we propose a missing value imputation model called UGAN-GRUD based on uncertainty matrix and time decay factor. UGAN-GRUD consists of two important components: UGAN, an improvement on traditional GAN, which includes a generator *G*, a discriminator *D*, and an uncertainty matrix *U*; and GRUD, an improvement on traditional GRU, which introduces the time decay factor. We conducted experimental studies, and the results show that UGAN-GRUD not only surpasses existing state-of-the-art methods in terms of imputation performance but also performs well in supporting subsequent classification and regression tasks.

The future research direction is to explore the interaction of correlated features [32–34] and their impacts on imputation performance. We believe that this will motivate new algorithm discoveries.

## Data Availability

The data and material that support the findings of this study are available on request from the corresponding author upon reasonable request.
